# Uncommon Variant of Type II Monteggia Fracture with Concomitant Distal Humeral Fracture

**DOI:** 10.1155/2015/374673

**Published:** 2015-10-15

**Authors:** Jihad F. Matta, George S. El Rassi, Hicham G. Abd El Nour, Rachel El Asmar

**Affiliations:** Department of Orthopedic Surgery and Traumatology, Saint Georges University Medical Center, Balamand University, P.O. Box 166378, Achrafieh, Beirut 1100 2807, Lebanon

## Abstract

Monteggia fracture-dislocation, a common injury sustained by pediatric population, is a rare entity in adults. It was first observed by Giovanni Battista Monteggia and later classified by Bado into 4 groups. The term “Monteggia equivalent or variant” was introduced to describe certain injuries with similar radiographic pattern and biomechanism of injury. Since then various types and their variants have been described in the literature. We present a complex fracture pattern in a 55-year-old male not previously described in the literature along with its treatment modality and favorable outcome.

## 1. Introduction

Monteggia fracture-dislocation is a rare entity in adults with an incidence of less than 1% [1]. It was first observed by Giovanni Battista Monteggia in 1814 and was described as a fracture of the proximal ulna with dislocation of the radial head [1–3]. In 1967, Bado classified “true Monteggia lesions” and classified them into 4 groups (Table 1). Bado also used the term “Monteggia equivalent or variant” to describe certain injuries with similar radiographic pattern and biomechanism of injury. Since then various types and their variants have been described in the literature [2]. We present a complex fracture pattern in a 55-year-old male not previously described in the literature that can be included under type 2 Monteggia variant, along with its treatment modality and favorable outcome.

## 2. Case Presentation

A 55-year-old male patient presented to the emergency department after sustaining a fall from 4-meter height. Patient attempted to break his fall and landed on his outstretched left hand. After the fall, the patient had severe pain and deformity of his left forearm. Initial assessment revealed a hemodynamically stable and cooperative patient but in distress due to severe pain. His left upper extremity had an obvious deformity in the forearm, with swelling and restricted range of motion due to pain. The patient also had a 3 cm wound on the dorsolateral side of his elbow.

**Figure 1:**
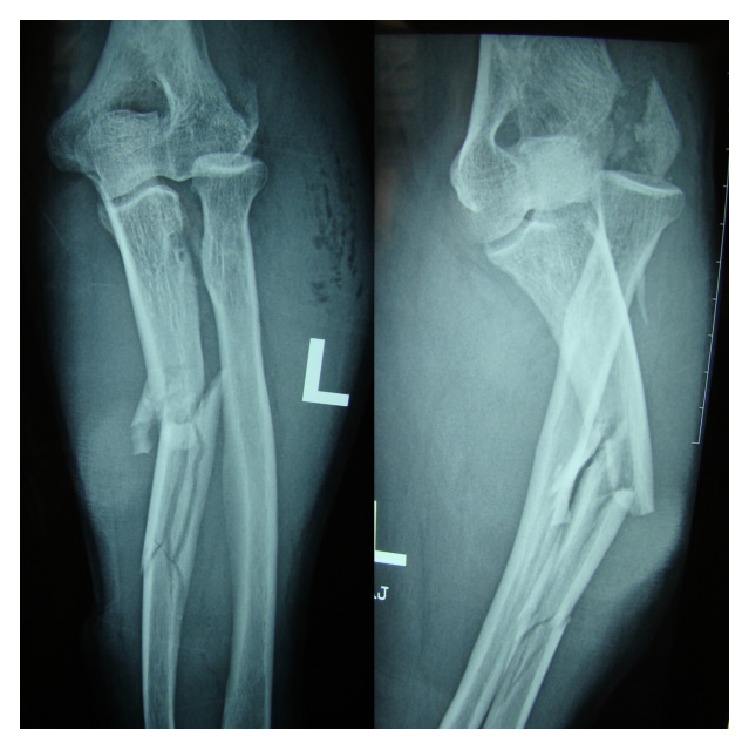
AP and lateral radiographs of the left forearm showing the Monteggia type 2 variant with fractured lateral humeral epicondyle.

**Figure 2:**
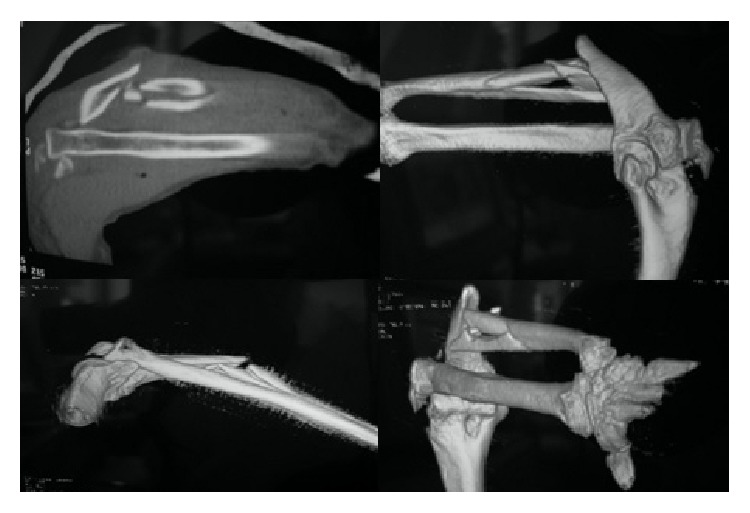
CT-scan with 3D reconstruction.

Roentgenograms showed a comminuted fracture of the ulnar shaft at the proximal and middle third junction with 35° of angulation along with a posteriorly dislocated nonfractured radial head and a fractured lateral humeral epicondyle (Figure 1). Furthermore due to the presence of the wound the fracture was classified as an open fracture. Computed topographic scan with 3D reconstruction was done to understand the injury more (Figure 2).

Patient was transferred the same day to the operative room for treatment. The wound was copiously irrigated with minimal debridement needed. Traction was first applied and it was sufficient to reduce the radial head and align the ulnar fracture. Open reduction of the ulnar fracture helped attain a better reduction and the fracture was fixed using a laterally applied 3.5 mm locked compressive plate (LCP) and ensuring a fixation of at least 6 cortices both proximally and distally. The fractured lateral humeral epicondyle was then reduced and fixed using 2 cannulated 3.5 mm partially threaded screws. The radial head stability was then evaluated and was found to be stable.

The postoperative period was uneventful and the patient was discharged home on day 3 postoperatively with a clean noninfected wound. The elbow was immobilized with a long posterior arm splint for 7 days. The elbow was then held in an articulated elbow brace and physical therapy was started. Active and passive range of motion exercises were started and at 6-week follow-up visit the patient was pain-free and showed full elbow extension, flexion to 140, supination to 90, and pronation to 80 degrees.

**Figure 3:**
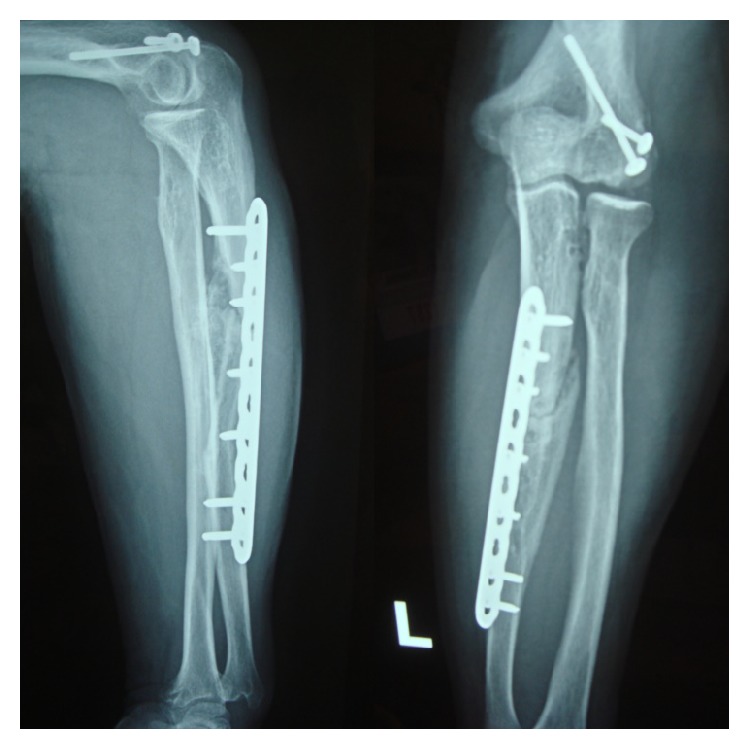
3-month follow-up X-ray revealing adequate healing.

**Figure 4:**
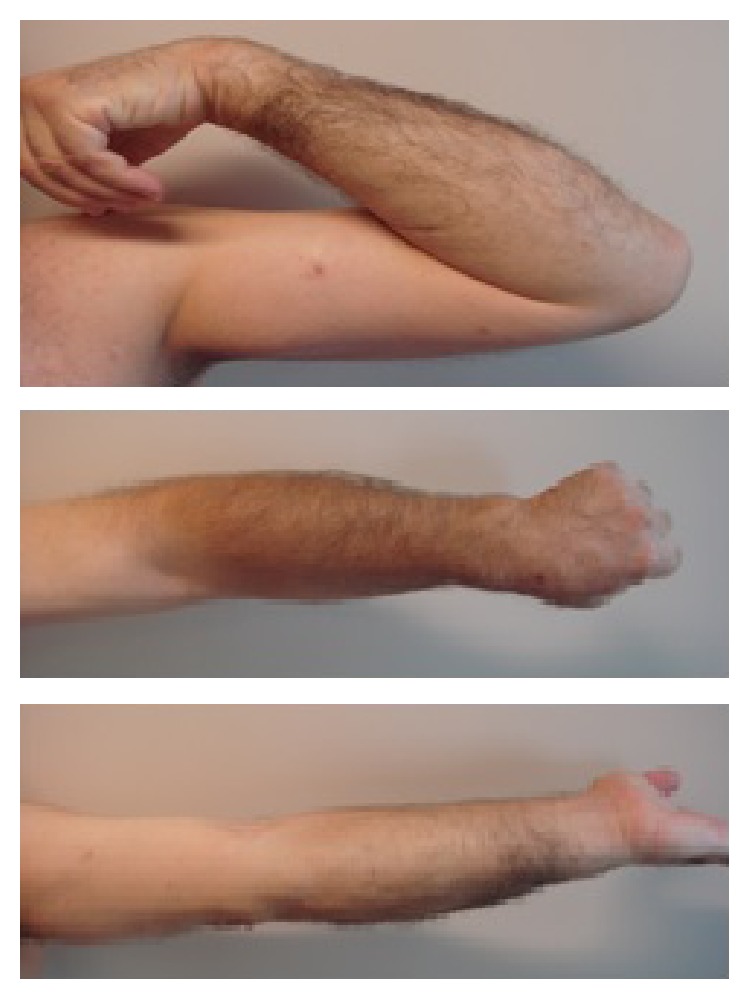
6-month follow-up visit revealing the resultant range of motion.

Radiographs done at 3-month follow-up visit revealed a healing ulnar and humeral fracture with a radial head in its adequate position (Figure 3). Patient at 6-month follow-up had full extension, flexion to 130°, supination to 90°, and pronation to 80° (Figure 4). Based on Broberg and Morrey scale [4], the end result was excellent.

## 3. Discussion

Our patient had Bado type 2 Monteggia variant that was an open fracture with concomitant ipsilateral distal humeral fracture, which represents a complex and intrinsically unstable fracture pattern. This complex high energy injury represents an uncommon Monteggia variant which was not previously described in the literature. The complexity of this injury comes from the fact that it is an intrinsically unstable fracture pattern associated with a distal humeral fracture and an open ulnar fracture and is rare in adults, mainly occurring in the pediatric population. The injury most probably resulted from elbow hyperextension with abducted arm in an attempt to break the fall. The impact on the elbow in extension would cause the olecranon to impinge on the humerus, levering the coronoid posteriorly beneath the trochlea, often with lateral rotation to cause failure of the posterolateral capsule and ligamentous support system [5].

Few cases have described posterior Monteggia lesions with concomitant ipsilateral distal humeral fracture. Arazi and Kapicioglu [6] reported a 13-year-old girl with Monteggia fracture-dislocation, nondisplaced distal radial fracture, and ipsilateral distal humeral fracture after a fall. Open reduction and internal fixation (ORIF) was performed for the distal humeral and ulnar fracture; closed reduction of the radial head was also performed. After one year of follow-up, the girl had full range of motion in her elbow joint. Our patient is an adult but had the same fracture-dislocation pattern except for the distal radial fracture. Beredjiklian et al. reported 2 cases with distal humeral fracture and ipsilateral anterior Monteggia fracture-dislocation [7]. The Monteggia lesion in our patient was posterior. Similar to our case, however, both of Beredjiklian's cases resulted from a high energy trauma and had an associated pelvic and lower extremity fractures. ORIF was done for the distal humeral and ulnar fracture on both cases with good results. Wang et al. presented a 19-year-old female with intercondylar distal humeral fracture and ipsilateral posterior Monteggia fracture-dislocation after a high energy motor-vehicle accident [1]. The patient had posterior interosseous nerve palsy but fully recovered after ORIF. Our patient had lateral epicondyle fracture as opposed to the intercondylar distal humeral fracture and no associated neurovascular injury.

## 4. Conclusion

Posterior Monteggia fracture-dislocation with ipsilateral distal humeral fracture is an extremely rare forearm injury with a high rate of complications. Early detection, full understanding of the injury, proper surgical intervention with strong internal fixation, and allowing early functional rehabilitation are essential for a favorable outcome.

## Figures and Tables

**Table 1:** Bado classification.

Type	Direction of radial head dislocation
Type 1	Anterior
Type 2	Posterior
Type 3	Lateral or anterolateral
Type 4	Anterior, with fracture radius shaft at the same level or distal to ulna fracture
